# Relationship of lower limb geometrics with femorotibial osteoarthritis in the toei cohort

**DOI:** 10.1038/s41598-022-16081-x

**Published:** 2022-07-19

**Authors:** Dung Huu Tran, Hironobu Hoshino, Yukihiro Matsuyama

**Affiliations:** Department of Orthopaedic Surgery, Hamamatsu University School of Medicine, 1-20-1 Handayama, Higashiku, Hamamatsu, Shizuoka 431-3192 Japan

**Keywords:** Bone, Bone quality and biomechanics

## Abstract

Osteoarthritis (OA) is a common disease of joints that is more common in females than in males. It is characterized by severe knee degeneration, damaged cartilage and appearance of osteophytes. Hip geometry and morphometric parameters in the knee joint vary between the sexes and depend on the severity of OA and the presence of osteophytes. Very few studies have assessed this phenomenon; therefore in this study, we assessed the difference in hip geometry and morphometric parameters in the lower limbs of males and females and evaluated the association of the differences with the presence or absence of osteophytes. Three hundred and six knees volunteers (104 male knees and 202 female knees) from the Toei region were included in the study between 2012 and 2018. The parameters measured were from the anteroposterior and lateral views of the hip and anteroposterior view of the knee. The participants were divided into 4 groups based on the assessment for osteophytes: Group 1 had no osteophytes, Group 2 had lateral compartment osteophytes, Group 3 had medial compartment osteophytes, and Group 4 had osteophytes in both compartments. Males had an increased femoral neck-shaft angle, femoral-tibial angle, and plateau angle, and a decreased femoral offset, femoral neck length, fibular-tibial angle, and knee rotation index than females. Group 2 had a greater height of the hip center, the angle between the femoral mechanical axis and the femoral shaft axis, condylar-hip angle, and plateau angle than Group 1. Group 3 showed an increased abductor angle, the angle between the femoral mechanical axis and the femoral shaft axis. Group 4 showed an increased angle between the femoral mechanical axis and the femoral shaft axis, femoral-tibial angle, and a decreased patellar shift index and knee rotation index. The angle between the femoral mechanical axis and the femoral shaft axis, condylar-hip angle, femoral-tibial angle, plateau angle, patellar shift index, and knee rotation index were different in both sexes and may be dependent on the presence or absence of osteophytes.

## Introduction

Osteoarthritis (OA) of the knee is a common disease in people over 60 years of age and is often complicated by severe cartilage damage^1^. It often occurs in a single compartment, and the medial compartment is the most commonly affected. This is because the load of the body on the knee joint is not uniform and has a medial predilection^2^. However, in females , the lateral joint space narrowing appeared with higher prevalance compared to male^3^. This difference in the sexes is explained by the differences in the morphometrics of the pelvis and the lower limbs between them^4^.

In women, hip abductor muscle weakness is lower than in men. This is besides the difference in the hip morphometry in the form of height of the hip center, lever arm ratio, femoral offset, etc., as related to the abductor muscle strength. The weakness of the abductor muscle may occur by itself (intrinsic) or by a change in the geometry of the pelvis and lower limbs (extrinsic)^4^. The anatomical geometry of the pelvis and lower limbs is measured based on the parameters of the lever arm ratio, femoral offset, angle between the femoral mechanical axis and the femoral shaft axis, and hip-knee-ankle angle among other parameters. The center of the femoral head serves as the fulcrum of the lever arm, and the force generated from the abductor muscle depends on the lever arm ratio. With age, the lever arm ratio decreases and the mechanical advantage of the abductors decreases, which may lead to abductor muscle weakness.

Femoral offset (FO) is the perpendicular distance from the femoral head center to the femoral shaft axis. A change in the FO changes the abductor moment arm and affects the abductor muscle force. An increase in the FO will increase the abductor muscle force, and a decrease in the FO will decrease the abductor muscle force^5^. Therefore, in hip arthroplasty, the surgeon increases the FO with the aim of increasing the abductor muscle force. This is done by using a femoral prosthesis that increases the offset^5^.

The abductor muscle influences knee joint load distribution through the movement of the pelvis in the frontal plane^6^. While walking, at the single-limb stance phase, the strong abductor muscles prevent the pelvis from moving excessively toward the opposite direction (the flexed leg). This ensures that the knee adduction moment does not change. Therefore, the force on the knee of the extended leg is balanced in both compartments. With weakness in the abductor muscle, the pelvis moves excessively towards the opposite direction (the flexed leg), the knee adduction moment increases, and the force exerted on the knee of the extension leg is distributed more to the medial compartment than to the lateral compartment. Therefore, weakness of the abductor muscle has a high relationship with the knee OA compartment.

Knee OA is classified according to the Kellgren and Lawrence system where osteophytes are not seen in grades 0 and 1 and are seen from grade 2 where cartilage damage commences. Previous studies have shown that the formation of osteophytes is driven by cytokine release from damaged cartilage and not by the mechanical forces on the joint capsule^7^. This explains why osteophytes are only found in severe knee OA from grade 2 of the Kellgren and Lawrence system. The osteophytes formed may not always appear in the same compartment of the knee^7^.

The knee adduction moment (KAM) indicates that the force exerted on the medial compartment of the knee joint follows the frontal plane^8^. Numerous studies have demonstrated that KAM is associated with compartment knee OA^9,10^. With weakness of the abductor muscle and excessive pelvic movement towards the opposite direction, the lateral structures of the knee such as the iliotibial band undergo excessive stress and the knee of the bearing leg tended to take a varus shape and undergo external rotation. This increases the KAM and increases the load on the medial compartment of the knee of the bearing leg affecting the knee rotation index and resulting in mechanical axis deviation.

With grade 2 medial compartment knee OA, osteophytes can be seen in the medial or/and lateral compartments. This research seeks to clarify if osteophyte formation has a relationship with hip geometry and morphometric parameters of the lower limb. Moreover, although it is well-known that the hip geometry and morphometric parameters in the lower limbs differ according to sex, the extent of the difference has not been fully explored. Therefore, this study has two aims:

1. Exploring the differences in hip geometry and morphometric parameters in the lower limbs of males and females.
2. Comparing the association of the differences in the hip geometry and morphometric parameters of the knee joint with the presence of osteophytes.

## Materials and methods

The study was conducted on 306 knees volunteers (104 male knees and 202 female knees) from the Toei region from 2012 to 2018. The age range was 59 to 84 years and 59 to 88 years for females and males, respectively. Radiography of the entire lower limb was performed in the anteroposterior view. Thereafter, the X-ray findings were used to grade the OA of the knee joint according to the Kellgren and Lawrence system: Group 1—knee OA grade 0 and grade 1, with no osteophytes; Group 2—knee OA grade 2 to grade 4, with lateral compartment osteophytes: Group 3—knee OA grade 2 to grade 4 with the medial compartment osteophytes; Group 4—knee OA grade 2 to grade 4 with osteophytes in both compartments.

Exclusion criteria for the study included: volunteers with congenital disease of the hip and knee joint; those that underwent replacement of the hip, knee, or ankle; those that underwent lower limb alignment or had a history of treatment of arthritis of the hip, knee, or ankle; those that had spinal deformity; and those that could not walk.

**Figure 1:**
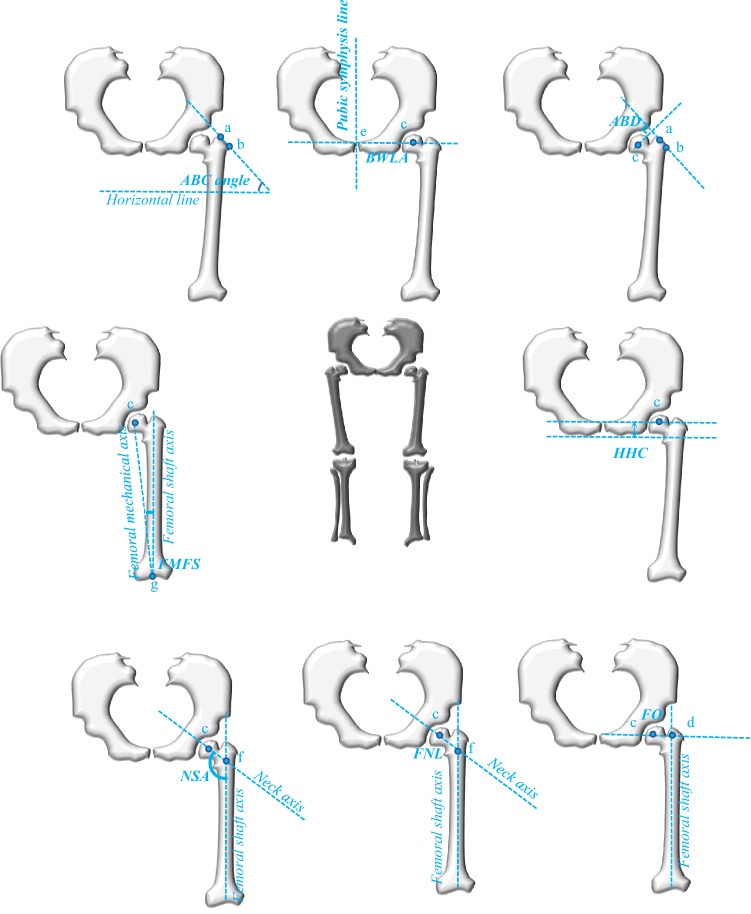
The hip geometry was measured on the anteroposterior view. *ABC* Abductor angle, *BWLA* body weight lever arm, *ABD* abductor lever arm, *HHC* height of the hip center, *FO* femoral offset, *FNL* Femoral neck length, *NSA* neck-shaft angle, *FMFS* angle between the femoral mechanical axis and the femoral shaft axis.

**Figure 2:**
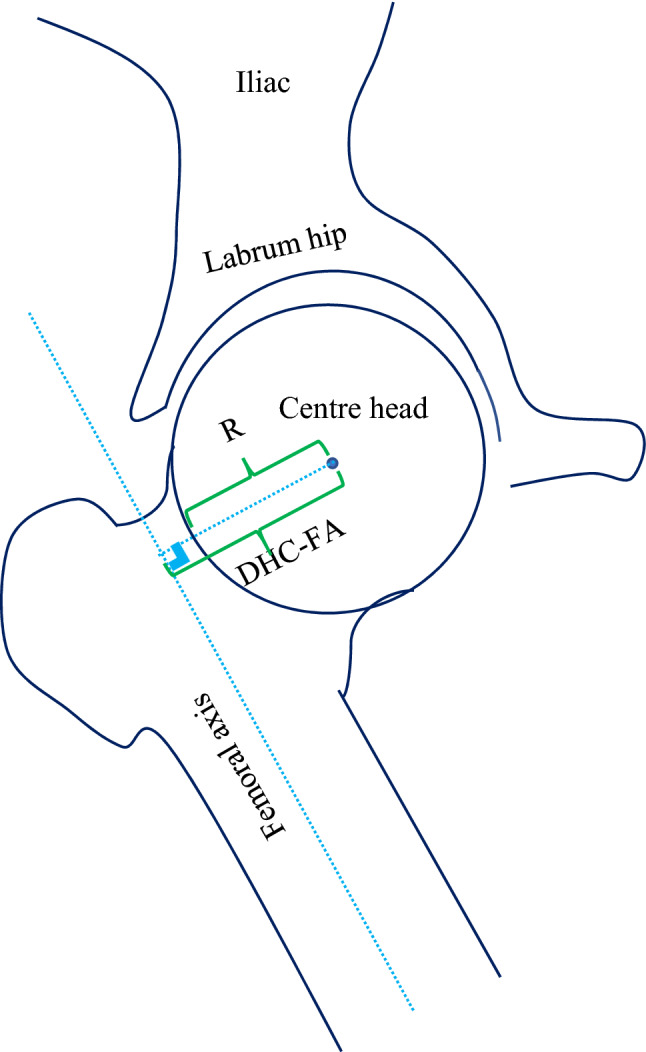
The hip geometry was measured on the lateral view. *R* radius of the femoral head, *D HC-FA* distance from the hip center to the femoral axis.

For the hip geometry and morphometric parameters in the knee joint, the OsiriX MD software (Pixmeo SARL, 266 Rue De Bernex, CH1233 Bernex, Switzerland) was used. The measurements were made using manually place landmarks by the author. The parameters were measured on the anteroposterior view of the hip, and they were determined as follows: body weight lever arm (BWLA) = the distance from the center of the femoral head (c point) to the pubic symphysis (segment ce); abductor lever arm (ABD) = the distance from the center of the femoral head (c point) to the line that follows the lateral aspect of the greater trochanter (ab line); lever arm ratio (LAR) = BWLA divided by ABD (LAR = BWLA/ABD)^11^; abductor angle (ABC angle) = the angle between the ab line and the horizontal line^11^; height of the hip center (HHC) = the distance from the center of the femoral head (c point) to the bottom of the ischium^11^; femoral head offset (FO) = the distance from point c to the femoral shaft axis^11^; femoral neck length (FNL) = the distance from point c to the intersection point between the neck axis and femoral shaft axis (corresponding to segment cf)^11^; and femoral neck-shaft angle (NSA) = the angle between the neck axis and femoral shaft axis; all these parameters were measured at the hip joint on the anteroposterior radiograph^11^. FMFS, the angle between the femoral mechanical axis and the femoral shaft axis^11^ (Fig. 1), was measured on the lateral view of the hip, and includes the following: R—radius of the femoral head and D HC-FA—distance from the hip center to the femoral axis (Fig. 2).

**Figure 3:**
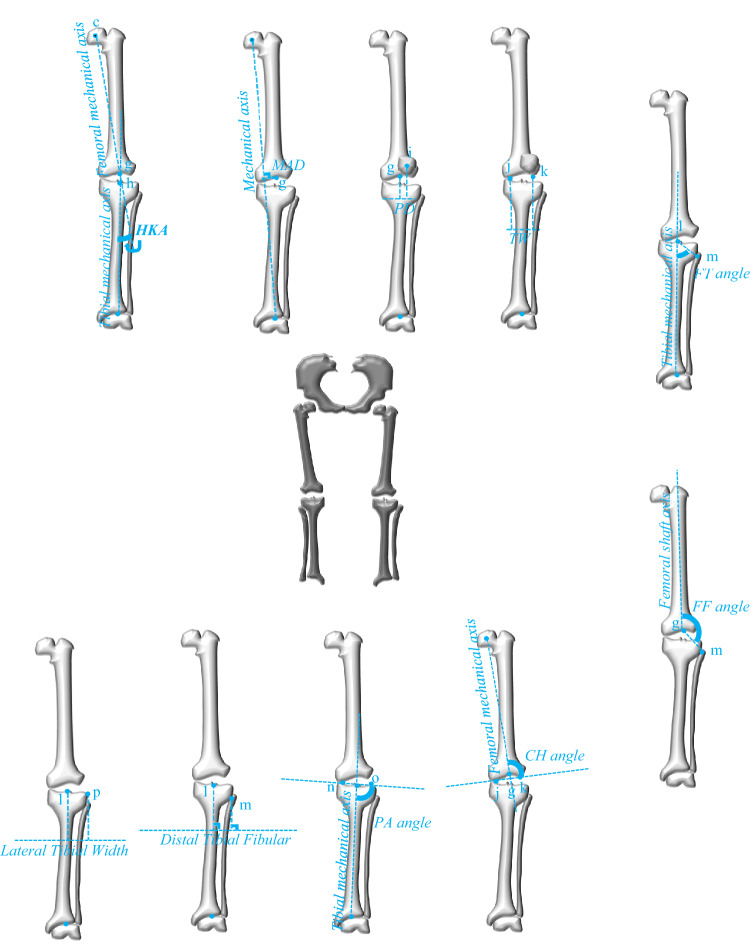
Morphometric parameters in the knee joint were measured on the anteroposterior view. *HKA* hip-knee-ankle angle, *MAD* mechanical axis deviation, *PD* patellar displacement, *TW* trochlear width, *FT angle* fibular-tibial angle, *FF angle* fibular-femoral angle, *CH angle* condylar-hip angle, *PA angle* tibial plateau-ankle angle.

For the knee, parameters measured on the anteroposterior radiograph include the following: hip-knee-ankle angle (HKA) = the angle between the femoral mechanical axis and the tibial mechanical axis; mechanical axis deviation (MAD) = the distance from the center of the knee (g point) to the mechanical axis; patellar displacement (PD) = the distance between a line that is perpendicular to line 1 and passing through the center of the knee, and a line that is perpendicular to line 1 and passing through the center of the patella^11^; trochlear width (TW) (lines 2 and 3 are parallel and exposed to the inside of the medial and lateral trochanter, and line 4 is connected to the bottom of the medial condyle with the lateral condyle and crossing points with lines 2 and 3, which are called the j and k points, respectively) = the width between j and k^11^; fibular-tibial angle (FT angle) = the angle between the tibial mechanical axis and the line from the center of the fibular (m point) to the center of the plateau (l point); femoral-fibular angle (FF angle) = the angle between the femoral shaft axis and the line from the center of the fibular (m point) to the center of the knee (g point); condylar-hip angle (CH) = the angle between line 4 and the femoral mechanical axis; plateau angle (PA) = the angle between line 5 and the tibial mechanical axis; tibio-fibular distance (TF) = the distance from the center of the fibular (m point) to the center of the plateau (l point); and lateral tibial width (LTW) = the distance from the center of the plateau (l point) to the outer limit of the lateral plateau (p point) (Fig. 3).

### Statistical analysis

SPSS software (version 23.0; IBM, NY, USA) was used for statistical analysis. When comparing the different parameters between females and males, binomial logistic regression, controlling for age, height, sacral slope, and knee deformity (normal, varus, and valgus), was used (Table 3). To assess the association between four-group dependent variable (the location of OA) and independent variables (the hip geometry and morphometric parameters of the knee joint), multinomial logistic regression was applied with the group 1 as the reference category (Table 4) and the age, sacral slope, height, knee deformity and sex were added as control variables.

### Ethical approval

The study was approved by the The Ethical Committee of the Hamamatsu University School of Medicine and performed in accordance with the ethical standards as laid down in the 1964 Declaration of Helsinki and its later amendments or comparable ethical standards.

### Informed consent

Informed consent was obtained from all participants included in the study.

## Results

For females, Group 1 had 67 knees (33.2%), Group 2 had 24 knees (11.9%), Group 3 had 57 knees (28.2%), and Group 4 had 54 knees (26.7%). For males, Group 1 had 47 knees (45.2%), Group 2 had 29 knees (27.9%), Group 3 had 18 knees (17.3%), and Group 4 had 10 knees (9.6%). In addition, based on the X-ray of the entire lower limb in the anteroposterior position, there were 18 and 13 normal (8.9% and 12.5%), 145 and 70 varus (71.8% and 67.3%), and 39 and 21 valgus (19.3% and 20.2%) cases for females and males, respectively.

Table 1 shows the characteristics of the study population.

**Table 1:** Characteristics of the study population.

Characteristics	Female				Male			
	N	Mean	SD	Min:Max	N	Mean	SD	Min:Max
Age	101	70.51	5.717	59:84	52	72.38	6.758	59:88
Height (cm)	101	150.67	5.620	136.30:164.40	52	162.77	6.346	148.80:178.30
Sacral slope (degree)	101	29.62	10.775	-4:55	52	31.79	8.410	13:58

*SD* standard deviation.

In females osteophyte appeared more in the medial compartment(28.2%), while in males, the lateral compartment was more popular (27.9%). For knee deformity, the varus deformity was the most popular for females and males (71.8% and 67.3%, respectively). The results are shown in Table 2.

**Table 2:** Characteristics of the knee joints in the study.

	Osteophyle position of the Knee					OA compartment of the knee					Deformity of the knee			
	Group 1 (Non) N (%)	Group 2 (Lateral) N (%)	Group 3 (Medial) N (%)	Group 4 (Both side) N (%)	N (%)	Group 1 (Non) N (%)	Group 2 (Lateral) N (%)	Group 3 (Medial) N (%)	Group 4 (Both side) N (%)	N (%)	Normal N (%)	Varus N (%)	Valgus N (%)	N N (%)
Male	47 (45.2%)	29 (27.9%)	18 (17.3%)	10 (9.6%)	104 (100%)	47 (45.2%)	12 (11.5%)	37 (35.6%)	8 (7.7%)	104 (100%)	13 (12.5%)	70 (67.3%)	21 (20.2%)	104 (100%)
Female	67 (33.2%)	24 (11.9%)	57 (28.2%)	54 (26.7%)	202 (100%)	67 (33.2%)	10 (5.0%)	62 (30.7%)	63 (31.2%)	202 (100%)	18 (8.9%)	145 (71.8%)	39 (19.3%)	202 (100%)

Table 3 shows the differences in the parameters of females compared with those of males. We used the binomial logistic regression controlling for age, height, sacral slope, and knee deformity. In males, there was decreased FO (OR 3.663 [95% CI 1.668–8.048]; p = 0.001), FNL (OR 2.717 [95% CI 1.258–5.870]; p = 0.011), FT angle (OR 1.093 [95% CI 1.025–1.166]; p = 0.007), and knee rotation index (OR 2909.94 [95% CI 84.285–100,465.122]; p = 0.000) compared with those in females. Furthermore, there were increases in the NSA (OR, 0.885 [95% CI 0.819–0.956]; p = 0.002), FF angle (OR 0.906 [95% CI 0.851–0.964]; p = 0.002), PA angle (OR 0.774 [95% CI 0.668–0.896]; p = 0.001) in males than in females.

**Table 3:** Different parameters of women compared to those of men.

Variable	Women		Men		Women compared to men		
	N	Mean (SD)	N	Mean (SD)	Adjusted OR	(95% CI)	p-value
ABC angle (degrees)	202	75.187 (5.352)	104	76.412 (4.137)	1.043	(0.948–1.148)	0.383
LAR	202	1.882 (0.203)	104	1.766 (0.174)	0.439	(2.347–20.378)	0.439
Height hip center (cm)	202	6.902 (0.503)	104	7.478 (0.642)	1.403	(0.610–3.227)	0.426
Femoral offset (cm)	202	3.188 (0.526)	104	3.164 (0.651)	3.663	(1.668–8.048)	0.001
FNL (cm)	202	4.427 (0.522)	104	4.462 (0.667)	2.717	(1.258–5.870)	0.011
Femoral NSA (degrees)	202	133.303 (6.183)	104	134.497 (5.463)	0.885	(0.819–0.956)	0.002
FMFS (degrees)	202	4.976 (0.747)	104	4.487 (0.799)	2.696	(1.528–4.757)	0.001
HKA angle (degrees)	202	3.491 (3.618)	104	2.910 (2.807)	0.973	(0.836–1.133)	0.727
MAD (cm)	202	0.999 (1.067)	104	0.890 (0.847)	0.880	(0.534–1.449)	0.615
Patellar shift index	202	0.246 (0.171)	104	0.364 (0.156)	0.003	(0.000–0.043)	0.000
FT angle (degree)	202	54.573 (5.697)	104	50.816 (7.540)	1.093	(1.025–1.166)	0.007
FF angle (degree)	202	137.817 (6.755)	104	141.959 (7.460)	0.906	(0.851–0.964)	0.002
CH angle (degree)	202	88.511 (2.856)	104	85.650 (2.314)	1.706	(1.374–2.119)	0.000
PA angle (degree)	202	94.603 (3.124)	104	96.490 (3.274)	0.774	(0.668–0.896)	0.001
Knee rotation index	202	0.799 (0.123)	104	0.650 (3.274)	2909.94	(84.285–100,465.122)	0.000

*ABC* Abductor angle, *LAR* Lever arm ratio, *FNL* Femoral neck length, *NSA* neck-shaft angle, *DHCFA/R* distance from the femoral head center to the femoral axis, divided by the radius, *FMFS* angle between the femoral mechanical axis and the femoral shaft axis, *HKA* hip-knee-ankle angle, *MAD* mechanical axis deviation, *FT* fibular-tibial angle, *FF* fibular-femoral angle, *CH* condylar-hip angle, *PA* tibial plateau-ankle angle, *OR* odds ratio, *CI* confidence interval.

Table 4 shows the association between the presence of OA and the hip geometry & morphometric parameters of the knee joint. The increase in Height hip center (OR 3.924, [95% CI 1.643–9.375]; p = 0.002), FMFS (OR 27.094, [95% CI 3.296–222.737]; p = 0.002), CH angle (OR 1.299, [95% CI 1.087–1.553]; p = 0.004) and PA angle (OR 1.392, [95% CI 1.13–1.714]; p = 0.002) resulted higher risk of the occurrence of lateral OA. Knees were more likely to have medial OA if it had a higher of ABC angle (OR 1.117, [95%CI 1.02–1.224]; p = 0.017) and higher FMFS (OR 35.654, [95%CI 5.024–253.032]; p < 0.001). The higher risk of both sides OA occurrence has been revealed in the higher FMFS (OR 93.406, [95%CI 7.979–1093.396]; p < 0.001), FT angle (OR 1.338, [95%CI 1.099–1.629]; p = 0.004). A higher Patellar shift index (OR 0.022, [95%CI 0–1.001]; p = 0.050) and Knee rotation index (OR 0.001, [95%CI 3.73E-07–0.873]; p = 0.046) showed a lower risk of having bot sides OA. Notably, knees that had high FMFS degree showed a relatively high risk of all types of OA.

**Table 4:** Comparison of parameters according to osteophyte compartment in the knee.

Variable	Group 2 vs Group 1 (Lateral OA vs No OA)			Group 3 vs Group 1 (Medial OA vs No OA)			Group 4 vs Group 1 (Both side OA vs No OA)		
	Adjusted OR	(95% CI)	p-value	Adjusted OR	(95% CI)	p-value	Adjusted OR	(95% CI)	p-value
ABC angle (degrees)	1.072	(− 0.969–1.186)	0.18	1.117	(1.02–1.224)	0.017	1.115	(0.993–1.252)	0.066
LAR	1.547	(–0.09–26.685)	0.764	5.233	(0.426–64.255)	0.196	4.083	(0.255–65.374)	0.320
Height hip center (cm)	3.924	(1.643–9.375)	0.002	1.98	(0.905–4.329)	0.087	1.256	(0.5–3.157)	0.627
Femoral offset (cm)	0.185	(0.005–6.699)	0.357	0.103	(0.003–3.713)	0.214	0.292	(0.005–17.605)	0.556
FNL (cm)	0.093	(0.006–1.568)	0.084	0.165	(0.012–2.293)	0.180	0.037	(0.002–0.879)	0.041
Femoral NSA (degrees)	1.249	(0.993–1.571)	0.057	1.167	(0.945–1.44)	0.151	1.262	(0.981–1.622)	0.070
FMFS (degrees)	27.094	(3.296–222.737)	0.002	35.654	(5.024–253.032)	0.000	93.406	(7.979–1093.396)	0.000
HKA angle (degrees)	1.233	(0.428–3.551)	0.698	0.703	(0.309–1.604)	0.403	1.89	(0.742–4.813)	0.182
MAD (cm)	0.296	(0.008–11.289)	0.512	7.267	(0.403–131.037)	0.179	0.342	(0012–9.932)	0.533
Patellar shift index	0.449	(0.026–7.831)	0.583	1.129	(0.097–13.212)	0.923	0.022	(0–1.001)	0.050
FT angle (degree)	1.025	(0.836–1.257)	0.814	0.906	(0.773–1.062)	0.223	1.338	(1.099–1.629)	0.004
FF angle (degree)	1.099	(0.86–1.403)	0.45	0.952	(0.789–1.149)	0.607	1.23	(0.984–1.537)	0.069
CH angle (degree)	1.299	(1.087–1.553)	0.004	1.062	(0.922–1.224)	0.406	0.887	(0.738–1.066)	0.200
PA angle (degree)	1.392	(1.13–1.714)	0.002	1.168	(0.981–1.392)	0.082	0.947	(0.76–1.179)	0.625
Knee rotation index	54.874	(0.146–20,690.423)	0.186	4.717	(0.022–1013.813)	0.571	0.001	(3.73E–07–0.873)	0.046

*ABC* Abductor angle, *LAR* Lever arm ratio, *FNL* Femoral neck length, *NSA* neck-shaft angle, *DHCFA/R* distance from the femoral head center to the femoral axis, divided by the radius, *FMFS* angle between the femoral mechanical axis and the femoral shaft axis, *HKA* hip-knee-ankle angle, *MAD* mechanical axis deviation, *FT* fibular-tibial angle, *FF* fibular-femoral angle, *CH* condylar-hip angle, *PA* tibial plateau-ankle angle, *OR* odds ratio, *CI* confidence interval.

## Discussion

The number of cases of knee OA in women was higher than in men, and the average age of women was lower than that of men(70.51 $\pm 5.717$ vs 72.38 $\pm 6.758$). The average height of women was also lower than that of men (150.67 $\pm 5.62$ vs 72.38 $\pm 6.758).$ Despite these variations, knee OA occurred more frequently in women than in men. Our previous research showed that older women are more associated with OA progression in knee joints^11^.

In the current research, the occurrence of medial compartment OA in both sexes was much higher than that of lateral compartment OA and OA in both compartments. This is similar to the findings of Ericsson et al.^12^. The research showed that the medial compartment osteophytes were higher than lateral compartment osteophytes or OA of both compartments in patients having medial compartment OA.

In this study, lateral compartment osteophytes were higher than medial or both compartment osteophytes in males, while medial compartment osteophytes were higher than lateral or both compartment osteophytes in females. However, for the total study group (males and females), medial compartment osteophytes were higher than lateral or both compartment osteophytes. Therefore, the result of the total study group was the same as that of Ericsson et al. The occurrence of osteophytes depends on many factors which are due to cytokine release from damaged cartilage in patients with severe knee OA, and this is more common in women. We suggest that the findings of males and females should be separated to get a more representative result as evidenced by the current study. The presence of osteophytes did not depend on OA location. In males, the medial compartment OA was the most common, but the lateral compartment osteophytes was the highest. In females, OA of both compartments was the most common, but the medial compartment osteophytes was the highest.

Similarly, hip parameters showed gender differences. FO and FNL were lower in males than in females, while NSA was higher in males than in females. HHC and ABC angle did not differ in terms of sex. After adjustment of the hip parameters for sex, age, height, sacral slope, and knee deformity, Group 2 showed an elevated increase in HHC compared with Group 1, and Group 3 showed an elevated increase in ABC angle than Group 1. Our previous research has been published to show that HHC changes are associated with OA progression in knee joints; however, FO was not found to be associated with OA progression in knee joints[11. In a study by Boissonneault et al., FO was shown to be related to the lateral compartment OA. An increase in HHC and decrease in FO reduces the power of the abductor muscle, resulting in decreased proximal control of the hip and uneven translation of the kinetics to the knee, worsening its OA progression. This study showed that increased HHC was associated with lateral compartment osteophytes or severe knee osteoarthritis, which appeared from grade 2 according to the Kellgren and Lawrence system. This was manifested by the occurrence of osteophytes. In the previous study, an increase in HHC was associated with OA, but we were unable to evaluate the OA occurring in the lateral compartment of the knee.

For FNL and NSA, although they differed between males and females, they did not differ among the groups. The increase in HHC and FNL in males had been shown in our previous research to be related to knee OA progression^11^. To explain this, the first reason was that the research method was the same, but the selected subject was different. The second reason was the difference in the number of males and females. The third reason was that the statistical tests used were different: in this research, binomial logistic regression and multinomial logistic regression were used to consider the impact of factors such as sex, age, height, sacral slope, and knee deformity. The final reason was the difference in sampling time.

Kinetically, the reduction in ABC angle is related to a more horizontal angle in the abductor muscles of the hip joint. A more horizontal angle in the abductor’s muscles will encourage internal rotation of the hip joint, anterior bundles of the gluteus medius and gluteus minimus have the main role^13^. Adduction moment in the knee is increased by excessive internal rotation of the hip joint. So, the increase of the ABC angle reduces the internal rotation of the hip joint. Adduction moment in the knee is reduced by reducing internal rotation of the hip joint. In this study, a higher ABC angle slightly increased the risk of exhibiting medial OA (Group 3 compared to Group 1). An increase in ABC angle is related to the OA process at the knee joint, and medial compartment.

The morphometric parameters in the knee joint were also shown to differ according to sex. After adjustment of the knee parameters for age, height, sacral slope, and knee deformity, the FT angle, CH angle and knee rotation index were found to be lower in males than in females, and the FF angle and PA angle were increased in males compared with those in females. Similarly, increasing FT angle was associated with an increased likelihood of occurring on both sides OA (Group 4 compared to Group 1), a severe valgus condition of the knee joint has increased FT angle and combined with internal rotation of the knee joint, has increased FT angle and decreased knee rotation index. The knee rotation index decreases because the internal rotation of the knee joint has increased the tibio-fibular distance. The increasing CH angle revealed a higher risk in Group 2 than in Group 1. To explain this result, a severe varus condition of the knee joint has increased CH angle. A severe varus condition combined with external rotation of the knee joint has been shown to be associated with medial compartment OA because it increases the KAM^9^. In the current study, medial compartment OA in the total study group was observed in 99 cases, which was higher than the number of cases observed with OA in the lateral or both compartments.

Lateral OA was more likely to occur in Group 2 if the PA angle was increased (compared to Group 1). This is because the severe OA status of the medial compartment indicates that the medial compartment plateau was damaged with cartilage loss, and the surface of the plateau has become eroded and concaved compared with the plateau of the lateral compartment.

FMFS angle increased in women, higher FMFS angle also increased the likelihood of OA in groups 2, 3 and 4 compared to group 1. The FMFS angle depends on the femoral mechanical axis and the femoral shaft axis, in which the femoral mechanical axis is more important than the femoral shaft axis. In knee replacement surgery, the study of Jeffrey J. Cherian et al. indicates the importance of lower limb mechanical axis restoration^14^. The increased FMFS angle affects the mechanical axis of the lower extremities and the balance of the knee joint is also affected.

PSI in men was higher than in women, PSI decreased when compared to the risk of OA between group 4 and group 1. The patella participates in the mechanism of the extension and flexion of the knee joint, ensuring the stability of the knee joint. Trochlear width (TW) in men is more common than in women, so the patellar displacement (PD) index is usually greater than that in women, PSI depends on TW and PD. The increase in TW decreases the PSI. Trochlear width (TW) increase also increases the patellar displacement (PD) index, and the movement of the patella along with the horizontal plane increases, It affects the mechanism of the knee extension and flexion and the balance of the knee joint.

The uniqueness of this study is that we considered several factors affecting the hip geometry and morphometric parameters of the knee joint such as sex, age, height, sacral slope, and knee deformity. Similarly, we also measured the corresponding parameters in the knee joint, whereas other studies merely measured the parameters in the hip or knee joint. The limitation of this study is that we did not conduct an evaluation of biological effects. Moreover, the study only evaluated volunteers in 2012, and it did not evaluate whether osteophytes will appear in another compartment over time. It is also not possible to assess the change in the parameters in the lower extremities over time.

## Conclusions

The FMFS was lower in men than in women and was related to lateral, medial, and both compartment osteophytes when compared to the group without osteophytes. In the same vein, the FT angle was lower in men than in women, and the PSI was higher in men than in women, they were related to osteophytes of both knee compartments when compared with the group without osteophytes.
